# Mitochondrial dysfunction drives natural killer cell dysfunction in systemic lupus erythematosus

**DOI:** 10.1172/jci.insight.195170

**Published:** 2026-01-29

**Authors:** Natalia Fluder, Morgane Humbel, Emeline Recazens, Alexis A. Jourdain, Camillo Ribi, George Tsokos, Denis Comte

**Affiliations:** 1 Department of Medicine, Division of Immunology and Allergy, Lausanne University Hospital, University of Lausanne, Lausanne, Switzerland.; 2 Department of Medicine, Beth Israel Deaconess Medical Center, Harvard Medical School, Boston, Massachusetts, USA.; 3 Department of Immunobiology, University of Lausanne, Epalinges, Switzerland.; 4 Department of Medicine, Division of Internal Medicine, Lausanne University Hospital, University of Lausanne, Lausanne, Switzerland.

**Keywords:** Autoimmunity, Immunology, Autoimmune diseases, Lupus, NK cells

## Abstract

Systemic lupus erythematosus (SLE) is a chronic autoimmune disease characterized by immune dysregulation and widespread inflammation. NK cells display marked functional impairment in SLE, including defective cytotoxicity and cytokine production, but the underlying mechanisms remain poorly defined. Here, we show that mitochondrial dysfunction and impaired mitophagy are key contributors to NK cell abnormalities in SLE. Using complementary structural, metabolic, and proteomic analyses, we found that SLE NK cells accumulate enlarged and dysfunctional mitochondria, exhibit impaired lysosomal acidification, and release mitochondrial DNA into the cytosol — features consistent with defective mitochondrial quality control. Transcriptional and proteomic profiling revealed downregulation of key mitophagy-related genes and pathways. These abnormalities correlated with reduced NK cell degranulation and cytokine production. We then tested whether enhancing mitochondrial quality control could restore NK cell function. The mitophagy activator Urolithin A improved mitochondrial and lysosomal parameters and rescued NK cell effector responses in vitro. Hydroxychloroquine partially restored mitochondrial recycling and reduced cytosolic mtDNA. These findings suggest that defective mitophagy and mitochondrial dysfunction are major contributors to NK cell impairment in SLE and that targeting mitochondrial quality control may represent a promising strategy for restoring immune balance in this disease.

## Abstract 2

This study links defective mitochondrial recycling to impaired natural killer cell function in SLE and identifies potential treatments to restore immune balance.

## Introduction

Systemic lupus erythematosus (SLE) is a multisystemic inflammatory autoimmune disease of unknown etiology, which mainly affects women of childbearing age. SLE is characterized by a breakdown in immune tolerance resulting in the production of autoantibodies, immune complexes, and autoreactive cells, which subsequently lead to organ damage (1). Although the contribution of different peripheral blood mononuclear cells (PBMCs; CD4^+^ T, CD8^+^ T cells, and B cells) to the pathogenesis of SLE has been widely examined, the precise mechanisms underlying tolerance breakdown remain elusive (2, 3).

NK cells represent a group of innate immune cells that play a pivotal role in the interface between the innate and adaptive immune system (4). These cytotoxic lymphocytes are essential for immune surveillance, recognition of healthy cells, and defense against viral infections and transformed cells. However, their contribution to autoimmune diseases like SLE remains poorly understood (4). Numerous studies have highlighted NK cell dysfunction in SLE, including reduced numbers in peripheral blood, impaired cytokine production (TNF-α, IFN-γ), reduced cytotoxicity, and defective antibody-dependent cellular cytotoxicity (ADCC) (5–8). These abnormalities may contribute to SLE disease pathogenesis by disrupting the ability of NK cells to control autoantibody-producing cells (9, 10). Additionally, compromised NK cell function may affect therapeutic responses, as monoclonal antibody treatments like rituximab rely on ADCC, a function mediated by functional NK cells (11, 12).

Emerging evidence points to mitochondrial dysfunction as an important contributor of immune cell dysregulation in SLE. T cells from patients with SLE exhibit abnormal mitochondrial metabolism, including increased mitochondrial mass (megamitochondria), mitochondrial hyperpolarization, and ATP depletion, which contribute to aberrant activation, oxidative stress, and inflammatory signaling — all central features of SLE pathogenesis (13, 14). Moreover, mitophagy, the mitochondrial quality control process, is impaired in SLE T cells, with the small GTPase RAB4A disrupting lysosomal degradation, leading to the accumulation of damaged mitochondria and persistent immune activation (13–17). Given the dependence of NK cell function on metabolic fitness, we hypothesized that similar mitochondrial abnormalities might contribute to NK cell dysfunction in SLE (13, 18). Additionally, we investigated whether enhancing mitochondrial quality control through pharmacological mitophagy activation could restore mitochondrial integrity and improve NK cell function in SLE.

In the present study, we performed a comprehensive analysis of the mitochondrial homeostasis in NK cells from patients with SLE, compared with matched healthy controls (HC). Our aim was to determine how mitochondrial abnormalities affect NK cell function and contribute to immune dysregulation in SLE. Furthermore, we explored the relationship between mitophagy defects and NK cell impairment, providing insights into the metabolic basis of NK cell dysfunction in SLE and potential therapeutic avenues to restore mitochondrial integrity.

## Results

### NK cells from patients with SLE accumulate enlarged dysfunctional mitochondria.

**Figure 1:**
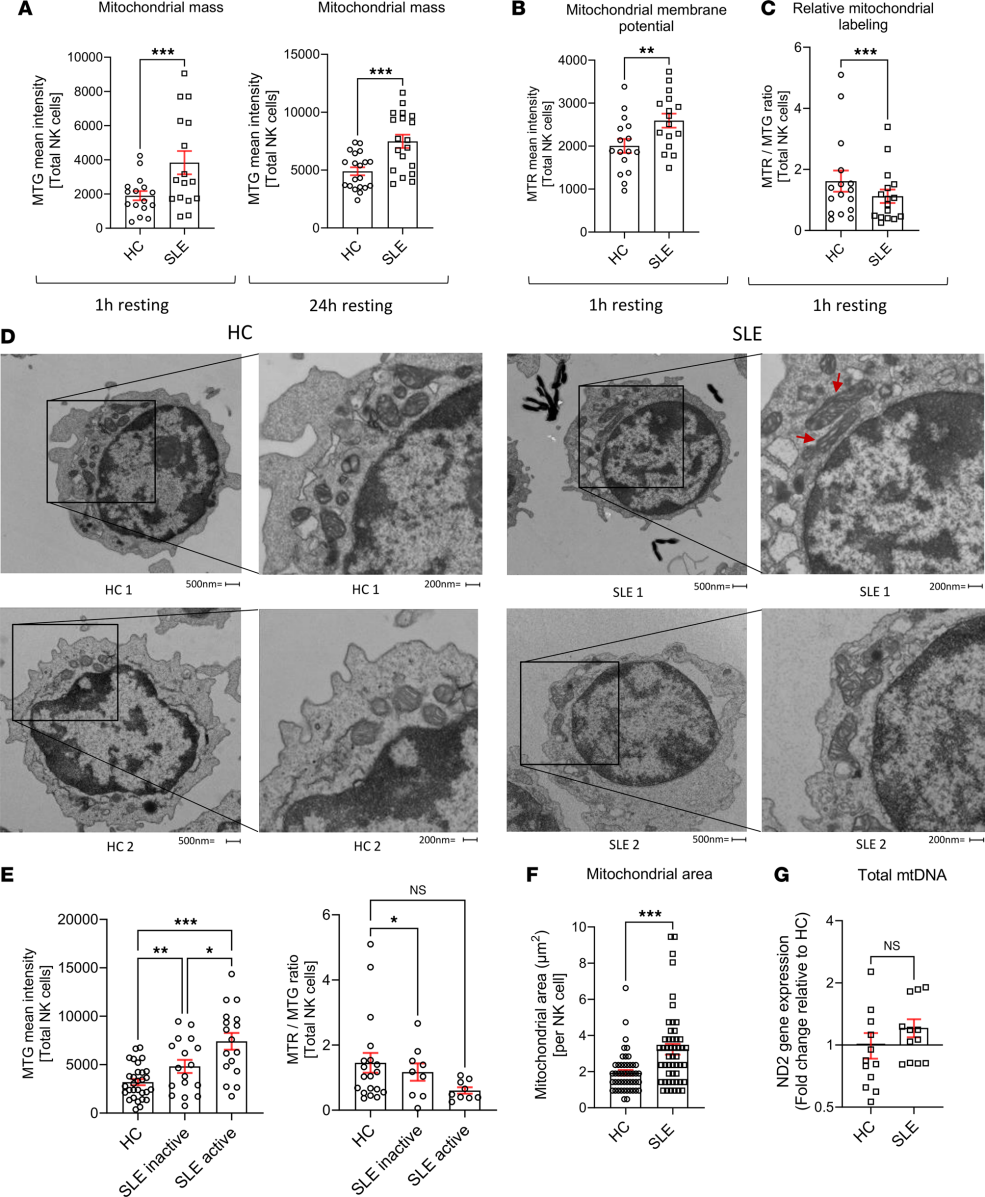
NK cells from patients with SLE accumulate enlarged dysfunctional mitochondria. (**A**–**C**) Mitochondrial mass, membrane potential, and relative mitochondrial labeling (MTR/MTG ratio) in NK cells from patients with SLE and HC, assessed at baseline by flow cytometry. (**D**) Mitochondrial mass and relative labeling stratified by disease activity score (SLEDAI). (**E**) Representative TEM images of NK cells from HC (*n* = 5) and patients with SLE (*n* = 5). Red arrows indicate enlarged mitochondria. Low-magnification images (left panels) show whole cells (scale bar: 500 nm); high-magnification images (right panels) show subcellular details (scale bar: 200 nm). (**F**) Quantification of mitochondrial area per NK cell (10 images per individual). Each symbol represents 1 NK cell. (**G**) Total mtDNA content quantified by qPCR (*n* = 12 per group). A log_2_ fold change > 0.5 or < –0.5 was considered significant; *P < 0.05, **P < 0.01, ***P < 0.001. Wilcoxon test (**A**–**C** and **F**), mixed-effects analysis (**E**), and Mann-Whitney test (**G**).

The numbers of NK cells are reduced in patients with SLE and exhibit impaired degranulation and altered cytokine production. Given that mitochondrial dysfunction has been demonstrated in SLE CD8^+^ T cells, we sought to investigate whether similar abnormalities contribute to NK cell dysfunction by assessing their mitochondrial fitness (9, 14, 19–21). Mitochondrial function was evaluated using flow cytometry to measure mitochondrial mass (MFI of MitoTracker Green [MTG]), membrane potential (MFI of MitoTracker Red [MTR]), and relative labeling (MTR/MTG ratio). SLE NK cells exhibited a significant increase in mitochondrial mass (Figure 1A) and mitochondrial membrane potential (Figure 1B), the latter reflecting the increase in mitochondrial content. However, normalization of membrane potential to mitochondrial mass revealed a reduction in relative mitochondrial labeling after 1hour of resting (Figure 1C). This ratio reflects mitochondrial membrane potential normalized to mitochondrial content, providing a relative indication of mitochondrial polarization per unit mass. This early time point was chosen to capture initial changes in mitochondrial function. To confirm the persistence of these changes, we reassessed mitochondrial mass in SLE NK cells, after resting for 24 hours. Although increased mitochondrial mass was observed in other PBMC populations such as B and T cells, this alteration was most pronounced in NK cells from patients with SLE compared with HC (Supplemental Figure 1A–D; supplemental material available online with this article; https://doi.org/10.1172/jci.insight.195170DS1). We next investigated whether mitochondrial mass alterations in SLE NK cells correlated with disease activity. Our cohort was divided into 3 groups: healthy, SLE inactive (Systemic Lupus Erythematosus Disease Activity Index [SLEDAI] ≤ 4), and SLE active (SLEDAI > 4) patients. Mitochondrial mass (MFI of MTG) and relative labeling (MTR/MTG ratio) were assessed across the groups. Mitochondrial mass was significantly increased in relation to disease activity, while mitochondrial relative labeling ratio showed a decreasing trend with increasing severity (Figure 1D). To determine whether these functional abnormalities correspond to structural changes, we performed transmission electron microscopy (TEM) analysis on NK cells from patients with SLE and HC. TEM revealed that the area occupied by mitochondria was significantly larger in SLE NK cells compared with controls (Figure 1, E and F). By analyzing 50 TEM images per group, we quantified both mitochondrial area and number. TEM confirmed an increase in mitochondrial size in SLE NK cells, while qPCR analysis showed that the mitochondrial genome remained the same, as indicated by the ratio of mitochondrial to genomic DNA (Figure 1G). These findings show that NK cells from patients with SLE accumulate enlarged and dysfunctional mitochondria, consistent with impaired mitochondrial clearance.

### Defective lysosomal acidification drives mitochondrial dysfunction in SLE NK cells.

**Figure 2:**
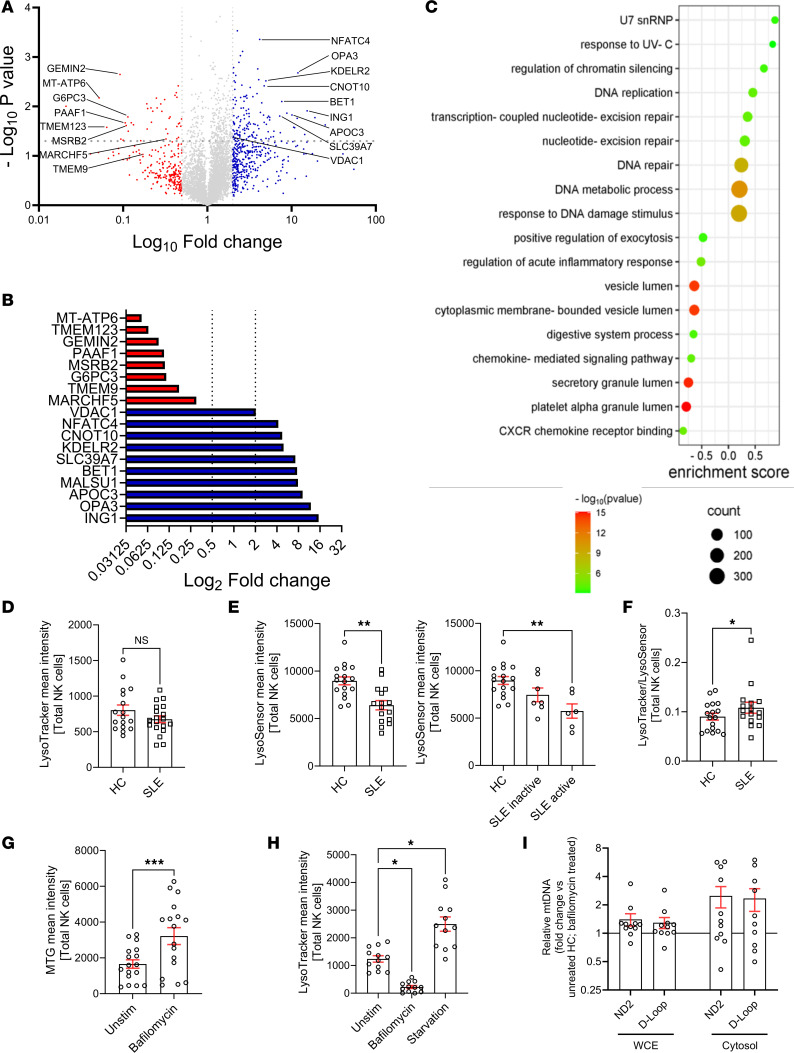
Defective lysosomal acidification drives mitochondrial dysfunction in SLE NK cells. (**A**) LC-MS–based proteomic analysis of NK cells isolated from healthy controls (HC, *n* = 4) and patients with SLE (*n* = 4). Volcano plot showing differentially expressed proteins in SLE versus HC NK cells (log_2_ fold change > 0.5 or < –0.5). (**B**) Selected significantly upregulated (red) and downregulated (blue) proteins in SLE NK cells (*P* < 0.05). (**C**) Gene ontology enrichment analysis of differentially expressed proteins. (**D** and **E**) Lysosomal number (**D**) and lysosomal pH (**E**, left panel) in NK cells from patients with SLE and HC assessed by flow cytometry using LysoTracker and LysoSensor probes, respectively. (**E**, right panel) Lysosomal pH stratified by disease activity score (SLEDAI). (**F**) Ratio of LysoTracker to LysoSensor fluorescence intensity in NK cells from patients with SLE and HC. *P < 0.05 and **P < 0.01 by Wilcoxon test (**D** and **F**) or mixed-effects analysis (**E**). (**G** and **H**) NK cells from HC were activated overnight in the presence or absence of bafilomycin A1 (100 nM) or subjected to starvation. Mitochondrial mass (**G**) and lysosomal number (**H**) were assessed by flow cytometry. Mitochondrial mass was analyzed using the Wilcoxon signed-rank test. Lysosomal number was analyzed using the Friedman test followed by Wilcoxon signed-rank post hoc tests. *P < 0.05, **P < 0.001. (**I**) NK cells from HC (*n* = 11) were activated overnight with or without bafilomycin A1 (100 nM) and fractionated into whole-cell extract (WCE), cytosolic, and mitochondrial fractions. Cytosolic mtDNA abundance was quantified by qPCR using ND2 and D-loop regions. Data are expressed as fold change in bafilomycin-treated cells relative to untreated HC samples. A log_2_ fold change > 0.5 or < –0.5 was considered significant.

To further investigate the mechanisms underlying mitochondrial dysfunction in SLE NK cells, we performed quantitative proteomic analysis focusing on proteins associated with mitochondrial homeostasis. Proteomic profiling revealed increased expression of proteins related to mitochondrial fragmentation (OPA3), suppression of mitophagy (SLC39A7), ER protein retention (KDELR2), negative regulation of NK cell function (NFATC4), mRNA degradation (CNOT10), activation of the NLRP3 inflammasome (APOC3), and metabolites flow through the mitochondrial channel (VDAC1). Conversely, we observed a reduction in proteins key for mitochondrial function (MT-ATP6), energy homeostasis in ER (G6PC3), mitochondrial clearance (MARCHF5), and cellular redox balance, involved in the switching-on of mitophagy (MSRB2) (Figure 2, A and B). Gene ontology (GO) enrichment analysis also revealed an upregulation of pathways involved in response to DNA damage and DNA metabolic process, along with a downregulation of genes associated with vesicle acidification and trafficking (Figure 2C). These findings pointed to potential disruptions in lysosomal function, a critical component of vesicle trafficking.

To investigate this, we assessed lysosomal trafficking and acidification in SLE NK cells using flow cytometry. Fluorescent probes specific for lysosomal number and pH revealed that, while the lysosomal number in SLE NK cells was comparable to HC (Figure 2D), lysosomal pH was significantly more alkaline in SLE NK cells (Figure 2E). Notably, lysosomal alkalinization increased in parallel with disease activity (Figure 2E), suggesting that lysosomal dysfunction becomes more pronounced in patients with higher disease activity. Consistent with these findings, the LysoTracker/LysoSensor ratio was significantly increased in SLE NK cells compared with controls (Figure 2F), indicating preserved lysosomal mass but disproportionate loss of acidification. To explore whether lysosomal acidification defects could drive mitochondrial abnormalities in health controls, we treated healthy NK cells with bafilomycin A1, a V-ATPase inhibitor. Bafilomycin A1 treatment resulted in increased mitochondrial mass (Figure 2G) and inhibited lysosomal acidification, reproducing the mitochondrial phenotype observed in SLE NK cells (Figure 2H). Moreover, bafilomycin A1 (100 nM) treatment led to the extrusion of mitochondrial DNA (mtDNA) into the cytosol of healthy NK cells after overnight incubation (Figure 2I). These findings indicate a link between defective lysosomal acidification and mitochondrial dysfunction in NK cells. Impaired lysosomal function likely contributes to the accumulation of enlarged, dysfunctional mitochondria, and probably the release of mtDNA, which may stimulate inflammatory pathways and the production of antibodies in SLE (17).

### NK cells from patients with SLE harbor functionally and structurally damaged mitochondria with an altered mitochondrial recycling.

**Figure 3:**
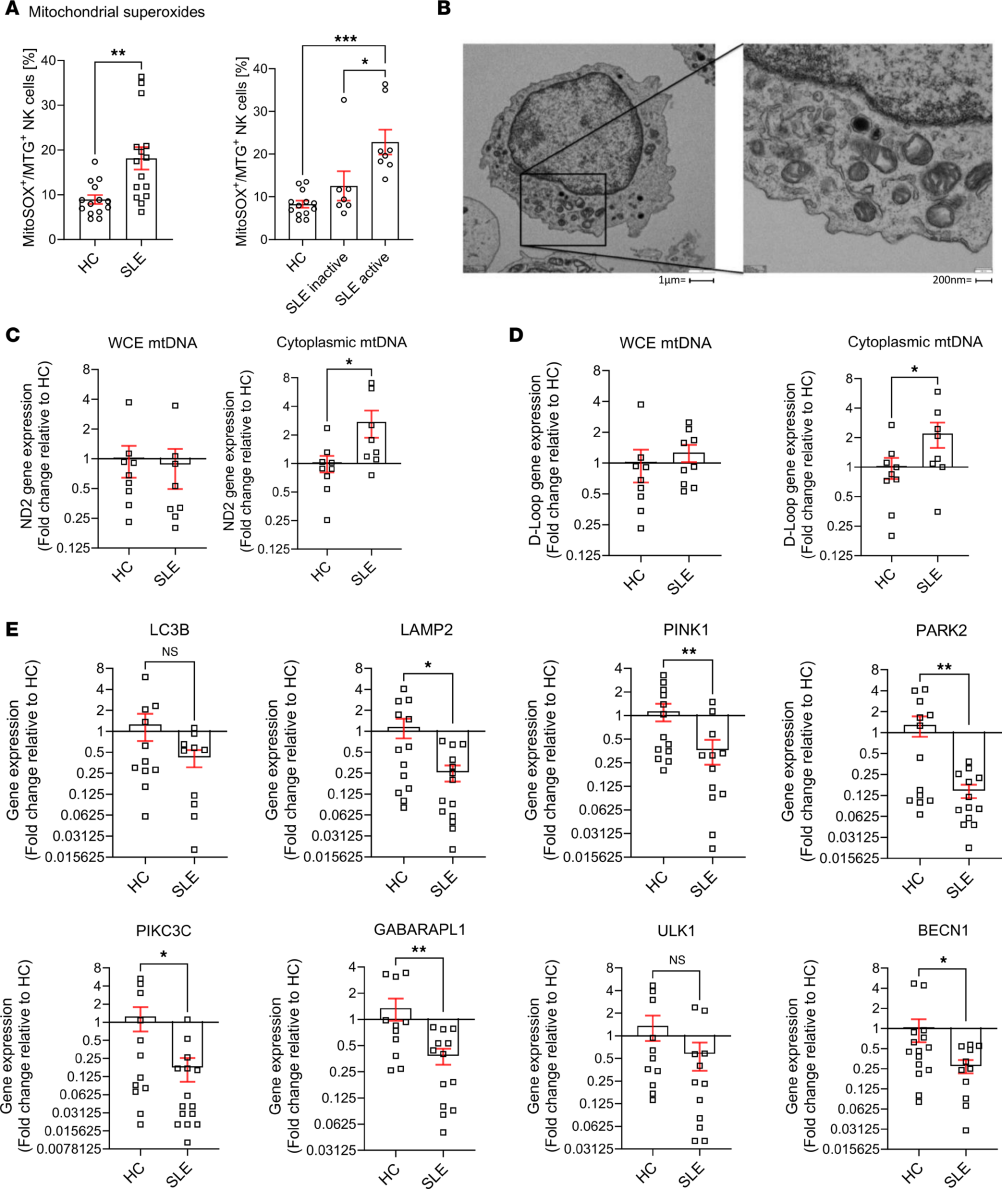
NK cells from patients with SLE harbor damaged mitochondria with disrupted ultrastructure and impaired mitophagy. (**A**) Mitochondrial super-oxide levels in NK cells from patients with SLE and healthy controls (HC) assessed by flow cytometry, based on the frequency of MitoSOX^+^/MTG^+^ cells (left panel) and stratified by disease activity score (SLEDAI) (right panel). *P < 0.05 by paired, 2-tailed Student’s *t* test (left panel) or by mixed-effects analysis (right panel). (**B**) Representative transmission electron microscopy (TEM) images of NK cells from patients with SLE showing mitochondrial ultrastructural abnormalities. Low-magnification image (left panel) shows whole cell (scale bar: 1 μm); high magnification image (right panel) shows subcellular details (scale bar: 200 nm). (**C** and **D**) NK cells isolated from HC (*n* = 10) and patients with SLE (*n* = 10) were subjected to subcellular fractionation. Relative mitochondrial DNA (mtDNA) abundance in whole-cell extracts (WCE), cytosolic, and mitochondrial fractions was quantified by qPCR using ND2 (**C**) and D-loop (**D**) regions. Data were normalized to the mean of HC samples. A log_2_ fold change > 0.5 or < –0.5 was considered significant; *P < 0.05 by Mann-Whitney *U* test. (**E**) Expression of mitophagy-related genes (*LC3B*, *LAMP2*, *PINK1*, *PARK2*, *PIKC3C*, *GABARAPL1*, *ULK1*, and *BECN1*) in NK cells from HC (*n* = 12) and patients with SLE (*n* = 12) assessed by qPCR and normalized to HC samples. A log_2_ fold change > 0.5 or < –0.5 was considered significant; *P < 0.05 and **P < 0.01 by Mann-Whitney *U* test.

Mitochondrial function is essential in maintaining a redox homeostasis where mitochondrial reactive oxygen species (mROS) play a central role in immune regulation. An unbalanced mROS production leads to apoptosis, insufficient cellular debris clearance, oxidative damage, diminished ATP production, and immune dysregulation characterized by skewing proinflammatory signaling in Th17 cells (20, 22). In our study, mitochondria from SLE NK cells displayed increased levels of superoxide (Figure 3A), predisposing them to mitochondrial damage, impaired debris clearance, and increased susceptibility to apoptosis. Notably, mitochondrial superoxide levels correlated with disease activity in SLE NK cells (Figure 3A). Electron microscopy showed alterations in the ultrastructure of the mitochondria of SLE NK cells, including mitochondrial cristae disorganization (Figure 3B). To ensure that these changes were not due to freeze-thaw artifacts, we compared freshly isolated and frozen NK cells from HC and found no significant differences in mitochondrial, lysosomal, or functional readouts (Supplemental Figure 3). Consistently, all TEM samples were processed immediately after thawing, and high-magnification images from HC confirmed preserved mitochondrial morphology compared with the abnormal mitochondria in SLE NK cells (Supplemental Figure 4). Given that mitochondrial cristae integrity is crucial for maintaining mtDNA compartmentalization and preventing inflammation, we hypothesized that these structural alterations could facilitate the release of mtDNA into the cytosol. To test this, we performed qPCR analysis to quantify the relative abundance of mtDNA in the cytosol and found significantly higher levels in SLE NK cells compared with HC (Figure 3, C and D), indicating increased mitochondrial membrane permeability and impaired compartmentalization of mtDNA. In parallel, proteomic analysis of SLE NK cells indicated pronounced disruptions in mitochondrial homeostasis and clearance mechanism. To further investigate these findings, we measured the expression of key mitophagy-related genes in NK cells from patients with SLE and HC using qPCR. Since lysosomal trafficking inhibition is known to induce the accumulation of enlarged mitochondria in healthy NK cells, we examined multiple stages of the autophagy pathway to evaluate the mitophagic flux in SLE NK cells. Our results demonstrate a significant reduction in the expression of genes essential for the PINK1-Parkin–dependent macroautophagy pathway, including, *LAMP2*, *PINK1*, *PARK2*, *PIKC3C*, *GABARAPL1*, and *BECN1* genes (Figure 3E). Other mitophagy-associated genes, including *ULK1* and ATG-related regulators, also displayed expression patterns consistent with impaired autophagic flux, further supporting defective mitochondrial quality control. Finally, as an exploratory metabolic assessment, we performed Seahorse analysis flux analysis. Glycolytic flux extracellular acidification rate (ECAR) was measurable in a limited subset of samples (Supplemental Figure 5) and showed no significant difference between patients with SLE and HC, suggesting that the metabolic abnormalities observed in SLE NK cells are not due to alterations in glycolysis. Unfortunately, OCR measurements could not be obtained due to low NK cell yields. Together, these findings reveal that SLE NK cells harbor mitochondria with both functional and structural damage, driven in part by impaired mitophagy. These defects were associated with the accumulation of dysfunctional mitochondria and increased mtDNA release.

### Pharmacological activation of mitophagy by Urolithin A restores mitochondrial integrity, lysosomal acidification, and NK cell function in SLE.

**Figure 4:**
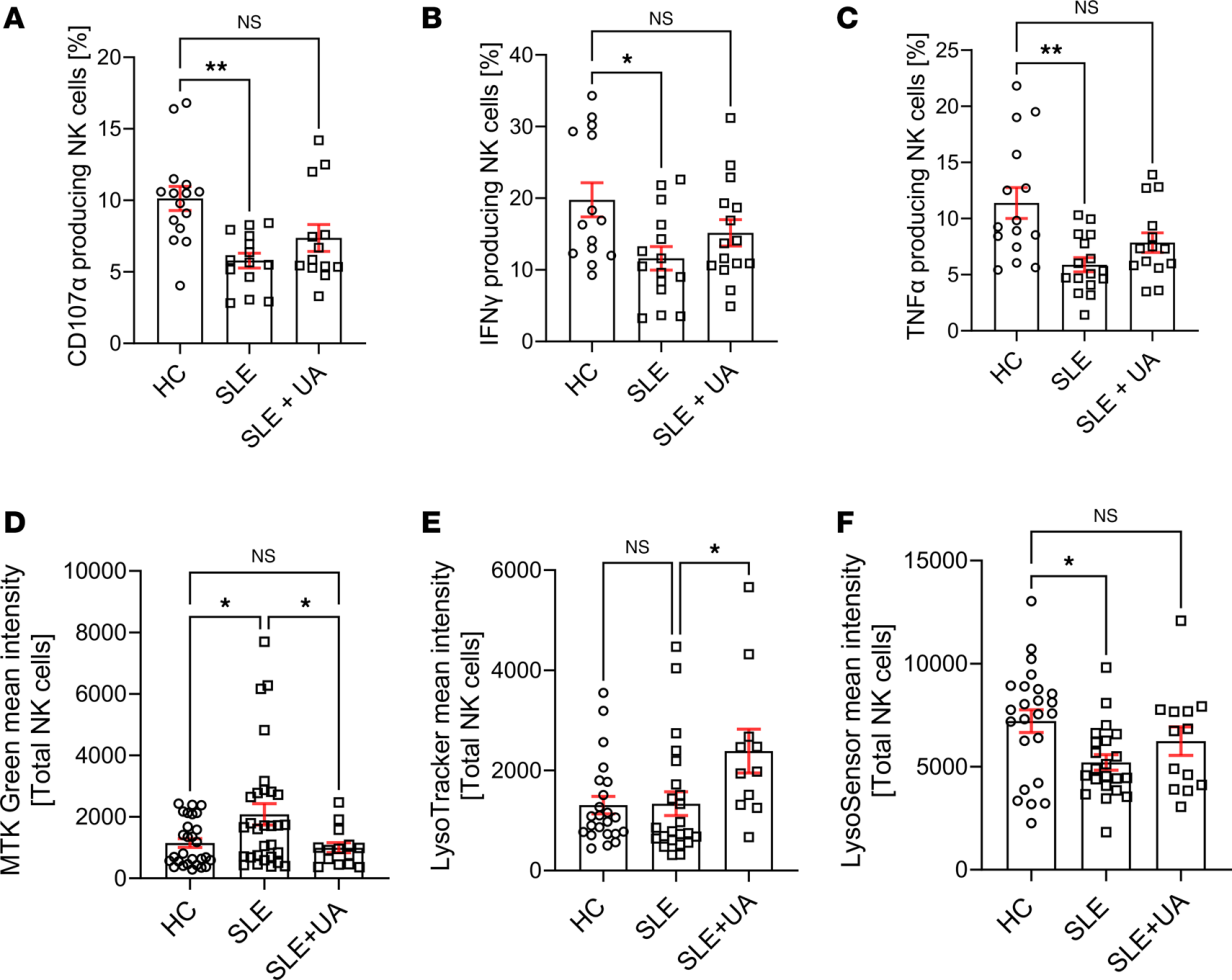
Pharmacological activation of mitophagy by Urolithin A (UA) restores mitochondrial integrity, lysosomal acidification, and NK cell function in SLE. NK cells from patients with SLE and healthy controls (HC) were activated overnight with IL-2 and IL-12 in the presence or absence of urolithin A (UA, 1 μM). (**A**–**C**) Degranulation and cytokine production were assessed by flow cytometry based on the frequency of CD107α^+^ (**A**), IFN-γ^+^ (**B**), and TNF-α^+^ (**C**) NK cells. (**D**) Mitochondrial mass in total NK cells assessed by flow cytometry. (**E** and **F**) Lysosomal number (**E**) and lysosomal pH (**F**) assessed by flow cytometry using LysoTracker and LysoSensor probes, respectively. Statistical significance was assessed using Kruskal-Wallis test (**A**–**C**, **E**, and **F**) or 1-way ANOVA (**D**). *P < 0.05, **P < 0.01.

We and others have previously demonstrated that NK cell function is severely impaired in SLE, characterized by defective production of IFN-γ and TNF-α and impaired degranulation, as indicated by decreased CD107a expression (6, 9, 23). To test whether restoring mitochondrial homeostasis could rescue NK cell function, we used Urolithin A, a natural mitophagy activator known to enhance mitochondrial clearance and improve cellular bioenergetics. This approach allowed us to assess whether improving endogenous mitochondrial turnover could functionally reverse NK cell dysfunction in SLE. SLE NK cells were activated overnight in vitro with IL-2 + IL-12. The addition of Urolithin A to the culture enhanced degranulation (CD107a) (Figure 4A), increased cytokine production (IFN-γ, TNF-α) (Figure 4, B and C), partially corrected mitochondrial mass (Figure 4D), and restored lysosomal acidification (Figure 4, E and F) compared with cells not exposed to Urolithin A. These findings indicate that improving mitochondrial quality control can restore NK cell effector functions in vitro.

### Hydroxychloroquine restores NK cell function in patients with SLE.

**Figure 5:**
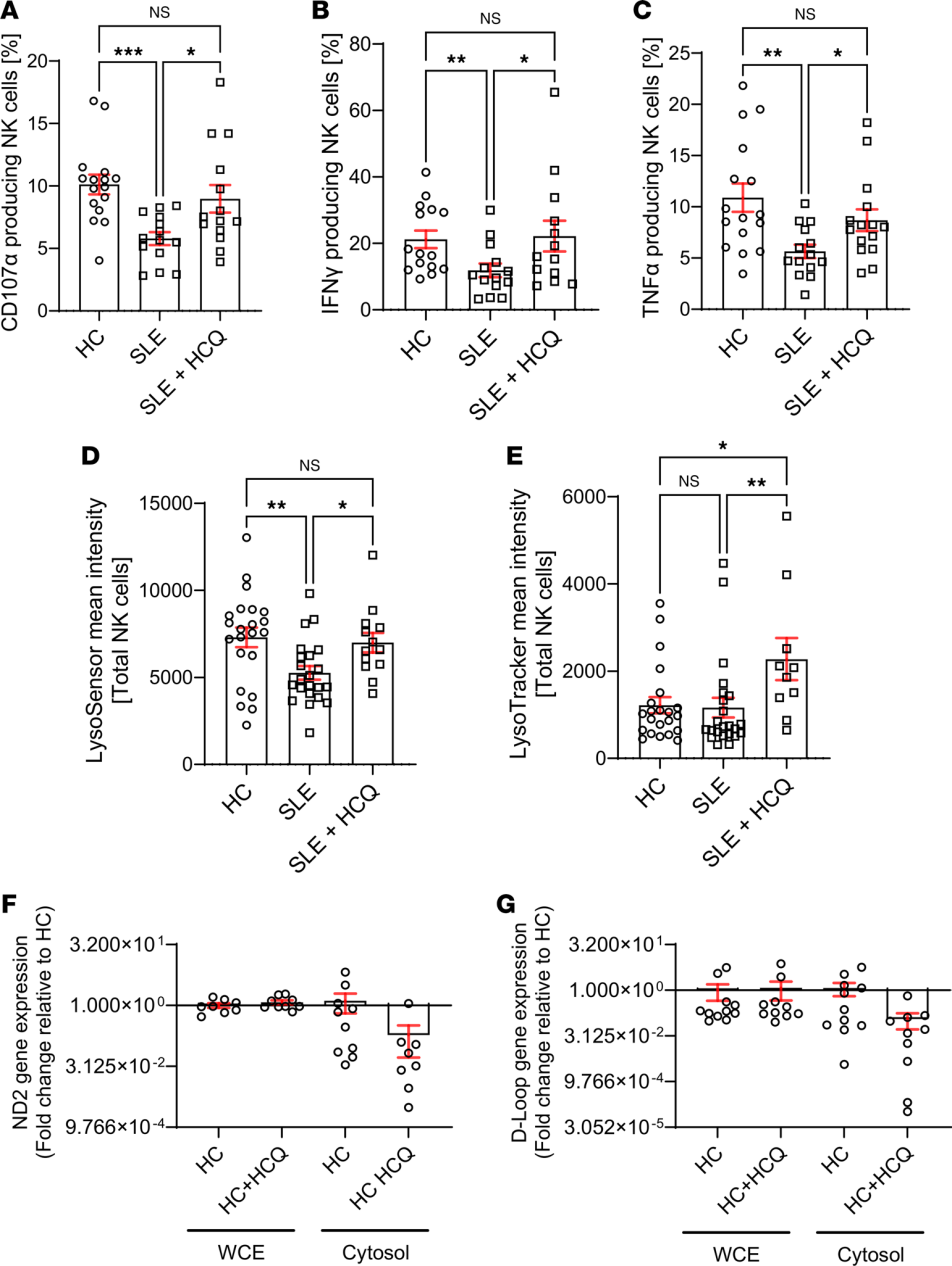
Hydroxychloroquine (HCQ) restores the cell function in NK cells from patients with SLE. NK cells from HC and patients with SLE were activated overnight with IL-2 and IL-12 in the presence or absence of HCQ (100 nM). (**A**–**C**) Degranulation and cytokine production of IFN-γ and TNF-α were assessed by flow cytometry based on the frequency of CD107α^+^ (**A**), IFN-γ^+^ (**B**), and TNF-α^+^ (**C**) NK cells. (**D** and **E**) Lysosomal pH (**D**) and lysosomal content (**E**) in NK cells from HC and patients with SLE assessed by flow cytometry using LysoSensor (**D**) and LysoTracker probes (**E**), respectively. (**F** and **G**) NK cells from HC (*n* = 10) were activated overnight with or without HCQ (1 mM) and subjected to subcellular fractionation. Cytosolic mitochondrial DNA (mtDNA) abundance was quantified by qPCR using ND2 (**F**) and D-loop (**G**) regions were normalized separately for each dataset. Statistical significance was assessed using Kruskal–Wallis test (**A**–**E**). ND2 gene expression (fold change relative to healthy control) was assessed in whole cell extracts (WCE) and cytosolic fractions. Within each fraction, HC and HC+HCQ conditions were compared using the Wilcoxon signed-rank test (**F** and **G**). *P < 0.05, **P < 0.01, ***P < 0.001. In vivo HCQ treatment status of patients with SLE at the time of sampling is reported in Supplemental Table 1.

SLE NK cells exhibit mitochondrial dysfunction, impaired mitophagy, and diminished effector functions. To explore whether hydroxychloroquine (HCQ), a widely used therapeutic in SLE, could modulate these cellular processes, we evaluated its effects on NK cell activation, mitochondrial recycling, and mitophagy-related gene expression (Supplemental Figure 2). In vitro treatment of activated (IL-2 + IL-12) SLE NK cells with HCQ reversed their reduced cytotoxicity and cytokine production, restoring these functions to levels observed in HC (Figure 5, A–C). To investigate whether HCQ could also address mitochondrial dysfunction, we examined its effect on lysosomal acidification. Our data show that HCQ increased lysosomal acidification in SLE NK cells (Figure 5, D and E). Moreover, we investigated the effect of HCQ on mtDNA release into the cytosol. HCQ treatment reduced the relative levels of cytosolic mtDNA in NK cells from HC, compared with untreated cells (Figure 5, F and G).

### HCQ restores the mitochondrial recycling in NK cells from patients with SLE.

**Figure 6:**
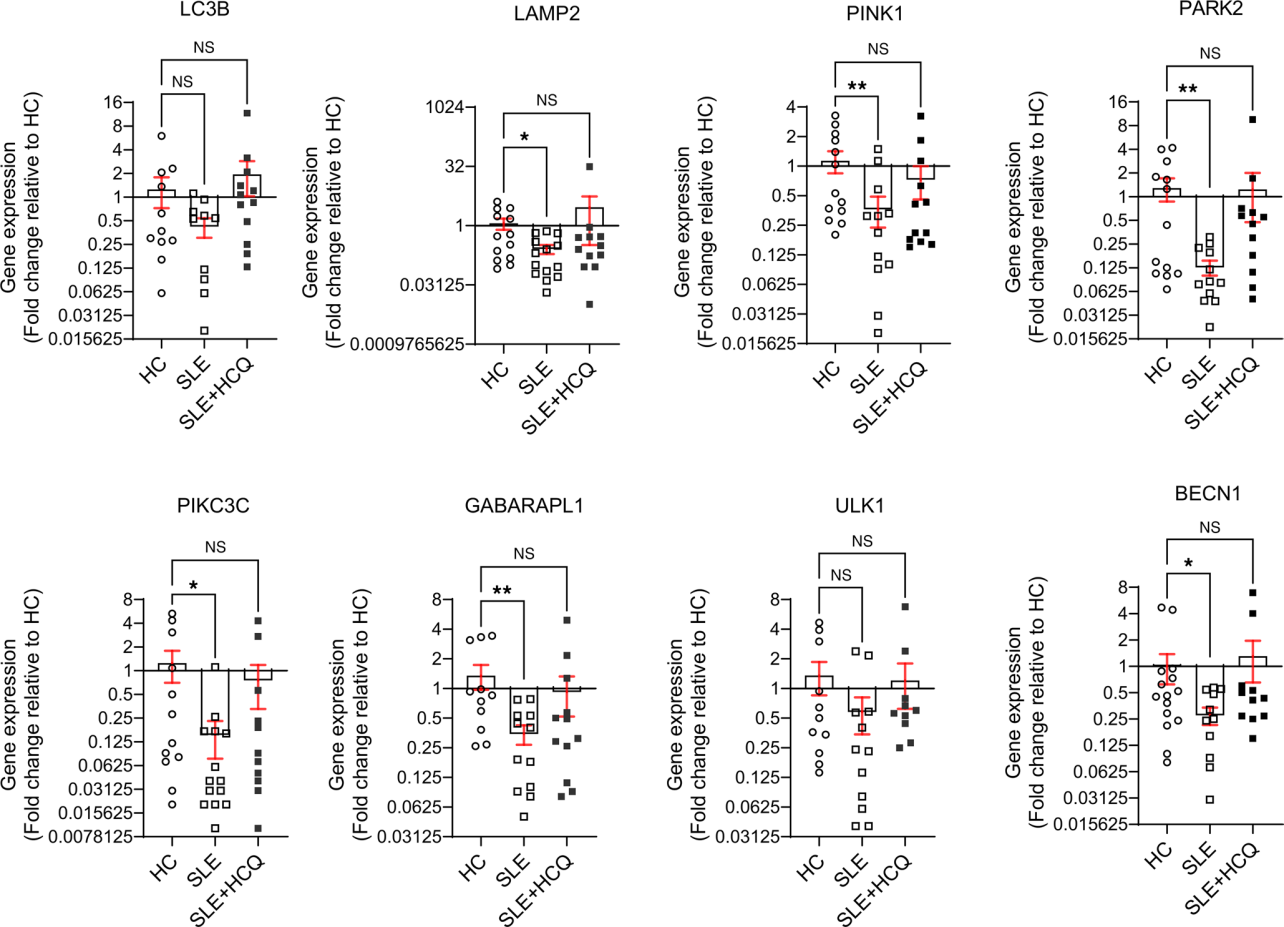
Hydroxychloroquine (HCQ) restores the mitochondrial recycling in NK cells from patients with SLE. NK cells from patients with SLE (*n* = 11) and HC were activated overnight with or without HCQ (1 μM), followed by qPCR analysis. Relative expression of mitophagy-related genes (*LC3B*, *LAMP2*, *PINK1*, *PARK2*, *PIKC3C*, *GABARAPL1*, *ULK1*, and *BECN1*) was assessed and normalized to HC samples. A log_2_ fold change > 0.5 or < –0.5 was considered significant; *P < 0.05 and **P < 0.01 by Kruskal-Wallis test. In vivo HCQ treatment status of patients with SLE at the time of sampling is reported in Supplemental Table 1.

Finally, we examined the effect of HCQ on the expression of key mitophagy-related genes. Overnight incubation with HCQ restored the expression of *LC3B*, *LAMP2*, *PINK1*, *PARK2*, *PIKC3C*, *GABARAPL1*, *ULK1*, and *BECN1* in SLE NK cells to levels comparable to those of HC (Figure 6).

## Discussion

NK cells are essential components of the immune system, playing key roles in antiviral defense, tumor surveillance, and immune regulation through cytotoxic activity and cytokine production (5). In SLE, NK cells exhibit profound dysfunction, including reduced cytotoxicity, impaired degranulation, and diminished cytokine production (e.g., IFN-γ and TNF-α) (6–8). These defects have important implications for SLE pathogenesis. First, the inability to eliminate virally infected and transformed cells increases susceptibility to infections and malignancies in patients with SLE (24). Second, the failure to clear autoreactive cells, such as antibody-producing plasma cells, CD4^+^ T cells, and dendritic cells, exacerbates autoantibody production and contributes to inflammation, immune complex deposition, and tissue damage (25–27). Third, NK cell dysfunction compromises ADCC, undermining the efficacy of monoclonal antibody therapies such as rituximab, particularly in severe manifestations including lupus nephritis (11, 12).

To elucidate the mechanisms underlying NK cell dysfunction in SLE, we focused on their immunometabolic regulation, with particular attention to mitochondrial fitness and recycling. Our findings reveal marked mitochondrial abnormalities in SLE NK cells, including increased mitochondrial mass, hyperpolarized membranes, and reduced mitochondrial labeling, indicative of impaired mitochondrial function. Additionally, defects in mitophagy, a critical mitochondrial quality control pathway, were observed, with SLE NK cells exhibiting reduced expression of genes involved in the *PINK1-Parkin*–dependent macroautophagy pathway (*LAMP2*, *PINK1*, *PARK2*, *PIKC3C*, *GABARAPL1*, *BECN1*) and impaired lysosomal acidification. Starvation is known to enhance lysosomal acidification through inhibition of mTOR and activation of TFEB, which promotes V-ATPase assembly and activity. This adaptive response facilitates autophagic degradation during nutrient deprivation and is consistent with our observation that lysosomal acidification can be modulated under stress conditions. It is important to note that NK cells are a heterogeneous population with distinct subsets and activation states that could influence mitochondrial characteristics. In a previous study from our group using single-cell mass cytometry (9), we demonstrated that SLE NK cells are reduced in number, display altered expression of multiple activation receptors, and exhibit impaired cytotoxicity and cytokine secretion.

Alterations in these pathways suggest a profound impairment in mitochondrial quality control. Reduced expression of *PINK1* and *PARK2* prevents efficient identification of dysfunctional mitochondria, allowing their prolonged accumulation within cells. The downregulation of *GABARAPL1*, *PIKC3C*, and *BECN1* disrupts autophagosome formation and cargo selection, impairing the initiation of mitophagy. Furthermore, insufficient *LAMP2* expression may lead to defective autophagosome-lysosome fusion, ultimately impairing lysosomal degradation of damaged mitochondria. These abnormalities result in the accumulation of dysfunctional mitochondria, leading to oxidative stress and the extrusion of mtDNA into the cytosol, a potent driver of inflammation through activation of cGAS and type I IFN responses, contributing to the amplification of autoimmune mechanisms. Consistent with this, our proteomic analysis revealed upregulation of VDAC1 in SLE NK cells. VDAC1 oligomerization has been shown to form pores in the outer mitochondrial membrane that facilitate mtDNA release into the cytosol, thereby driving type I IFN responses and lupus-like disease (28). This mechanism provides a plausible link between increased VDAC1 expression and the enhanced cytosolic mtDNA detected in SLE NK cells, further reinforcing the role of mitochondrial pore-mediated mtDNA release in immune dysregulation.

Collectively, these findings support the concept that mitochondrial dysfunction and impaired mitophagy are central contributors to NK cell abnormalities and broader immune dysregulation in SLE. The mitochondrial abnormalities in SLE NK cells result from converging defects in quality control pathways, including impaired *PINK1-PARK2*– and *BECN1*-dependent mitophagy, defective lysosomal acidification limiting autophagosome clearance, and increased VDAC1-mediated pore formation. Similar mitochondrial dysfunction has also been reported in SLE T cells, B cells, and monocytes, where defective clearance of damaged mitochondria contributes to oxidative stress, mtDNA release, and type I IFN activation. Our findings therefore extend these shared pathogenic mechanisms to NK cells, a population in which mitochondrial quality control is directly linked to cytotoxicity and cytokine production, thereby amplifying immune dysregulation in SLE.

The ability of Urolithin A to restore mitochondrial integrity and NK cell function indicates that enhancing mitochondrial quality control is sufficient to rescue NK cell effector functions. This supports the hypothesis that defective mitochondrial recycling, rather than intrinsic NK cell signaling defects, contributes substantially to their dysfunction in SLE.

HCQ is widely used in SLE to reduce disease activity, prevent flares, and decrease corticosteroid dependency, with additional benefits such as thrombotic risk reduction and improved pregnancy outcomes (21, 22). At the molecular level, HCQ accumulates in lysosomes, modulating pH and affecting key processes like autophagy, antigen presentation, and cytokine production. While high doses (5–50 μM) can impair lysosomal function and induce toxicity, our study demonstrated that low, clinically relevant, doses (0.1–1 μM) effectively restore NK cell function without cytotoxic effects. Given HCQ’s multifaceted mechanisms of action, it is unclear whether its observed effects on mitochondrial function are direct or secondary to its modulation of lysosomal activity and immune signaling. Additionally, a significant proportion of patients in our cohort was already receiving HCQ treatment, raising the possibility that any in vitro effects may not fully replicate in vivo responses. While our results suggest that HCQ partially restores mitochondrial homeostasis and reduces mtDNA release, further studies will be needed to determine whether these effects are due to direct modulation of mitophagy or secondary to its immunomodulatory properties. Taken together, our data indicate that HCQ modulates NK cell effector functions in parallel with mitochondrial and autophagy-related pathways in SLE, although the extent to which these effects are mechanistically linked remains to be determined.

Several limitations should be acknowledged. First, the cross-sectional nature of this study limits our ability to determine whether mitochondrial dysfunction is a primary driver of NK cell impairment in SLE or a secondary consequence of chronic immune activation. Future studies should include longitudinal analyses to better define the temporal relationship between mitochondrial abnormalities and NK cell function. Second, while our findings suggest an impairment in mitophagy, our study primarily relied on gene expression analyses to assess autophagy-related pathways. Given that autophagy is a dynamic process, further studies assessing protein markers such as LC3B and p62/SQSTM1, as well as functional mitophagy flux assays, will be needed to confirm these findings. In particular, we were unable to perform protein-level analyses of LC3 lipidation due to limited NK cell material; instead, our proteomic data provided indirect evidence of mitophagy imbalance, with downregulation of key regulators alongside compensatory increases in LC3 family proteins. Similarly, for transcriptional changes shown in Figure 6, we did not validate each target at the protein level, although our proteomic and functional data support the overall directionality of these findings. Third, although our findings indicate mitochondrial dysfunction, our metabolic analyses were exploratory. Seahorse extracellular flux assays provide valuable insights into cellular bioenergetics; however, their feasibility in primary NK cells isolated from PBMCs is technically challenging. In a subset of samples, glycolytic flux (ECAR) was successfully measured and showed no significant difference between SLE and healthy NK cells, suggesting that glycolytic pathways remain largely preserved. In contrast, OCR measurements could not be reliably obtained due to low NK cell yields, preventing a comprehensive assessment of mitochondrial respiration. These technical constraints are inherent to studies involving primary NK cells isolated from patient peripheral blood, where cell numbers are often limited. Fourth, as all experiments were performed on thawed PBMCs, we specifically controlled for this by comparing fresh and frozen samples and by providing HC TEM images, which support the robustness of our approach. We recognize that frozen PBMCs may exhibit metabolic alterations that could influence mitochondrial readouts. However, given that both SLE and HC NK cells were processed under identical conditions, we believe that the observed differences are biologically meaningful. Nevertheless, future studies using freshly isolated NK cells will be required to fully validate these findings. Fifth, we emphasize that bafilomycin A1 was used as a pharmacological model to mimic lysosomal alkalinization, but it does not fully recapitulate SLE pathology; its effects on mitochondrial dynamics may also result from inhibition of lysosomal degradation rather than direct disease-specific mechanisms.

In conclusion, our study identifies mitochondrial dysfunction and impaired mitophagy as central contributors to NK cell abnormalities in SLE. These defects contribute to oxidative stress, mtDNA release, and chronic immune activation, providing mechanistic insight into how NK cell metabolism influences disease pathogenesis. While HCQ appears to partially restore mitochondrial homeostasis, its precise mechanism of action in this context remains unclear. Future research should explore targeted approaches to modulate mitochondrial quality control mechanisms in NK cells, which may provide therapeutic strategies to restore immune balance and improve clinical outcomes in SLE.

## Methods

Supplemental Methods are available online with this article.

### Sex as a biological variable

Both female and male participants were included in this study. The cohort showed a predominance of female participants (88.5%), reflecting the known epidemiology of SLE. HC were matched to SLE participants by sex, ethnicity, and age. Given the limited number of male participants, analyses were not powered to formally assess sex-specific effects, and results were therefore analyzed across the full cohort.

### Study design

This observational cross-sectional study aimed to analyze mitochondrial homeostasis in NK cells from patients with SLE compared with matched HC. The objective was to elucidate how immunometabolic abnormalities contribute to NK cell dysfunction and SLE pathogenesis. A total of 104 patients with SLE were included based on the 1997 revised American College of Rheumatology (ACR) classification criteria and/or the Systemic Lupus International Collaborating Clinics (SLICC) criteria (29, 30). For each SLE participant, an HC was selected to match sex, ethnicity, and age within a ± 10-year range. Patients and HC were enrolled from the Division of Immunology and Allergy at Centre Hospitalier Universitaire Vaudois (CHUV). All participants, including HC, were part of the Swiss Systemic Lupus Erythematosus Cohort Study (SSCS) (31). Because NK cell numbers obtained from patient samples were often limited, not all assays could be performed on every individual. Consequently, different subsets of patients with SLE (or, when applicable, different blood-draw time points from the same patient) were used across experiments depending on NK cell availability. Characteristics of the patients with SLE included in this study are provided in Table 1 and Supplemental Table 1.

#### PBMC isolation and cell culture.

PBMCs were enriched by density gradient centrifugation FICOLL 400 (Merck) at 300*g* for 30 minutes at room temperature, brake off. The PBMCs were cryopreserved in liquid nitrogen for downstream analyses. Cells were cultured in RPMI (Gibco; Life Technologies) containing 10% heat-inactivated fetal bovine serum (FBS; Institut de Biotechnologies Jacques Boy), 100 IU/mL penicillin and 100 μg/mL streptomycin (Bio Concept), referred to as complete RPMI (cRPMI).

### Flow cytometry

PBMCs from patients with SLE and HC were thawed in RPMI containing 20% FBS, centrifuged at 300*g* for 10 minutes at room temperature resuspended in cRPMI, and pelleted. Cells were stained with Live/Dead-APC-H7 or PB (Invitrogen; catalog nos. L34994 and L34962) and cell surface antibodies: CD3-BUV737 (clone UCHT1; BD Biosciences; catalog no 612750) or APC-H7 (clone SK7; BD Biosciences; catalog no 560176), and CD56-BUV395 (clone NCAM16.2; BD Biosciences; catalog no 563554), in parallel to different fluorescent probes specific to mitochondrial fitness, superoxides and lysosomal acidification.

#### NK cell mitochondrial fitness.

PBMCs were incubated for 20 minutes at 37°C with the cell-permeable fluorescent probes MTG and MTR (Invitrogen). MTG measured mitochondrial mass, while MTR assessed the mitochondrial membrane potential (ΔΨ_m_). Cells stained with MTR were fixed BD CellFIX before analysis on LSR Fortessa (BD Bioscience).

#### NK cell mitochondrial superoxides.

PBMCs were incubated for 15 minutes at 37°C, in presence of the MitoSOX fluorescent probe (Invitrogen). PBMCs stained with MitoSOX were analyzed on LSR Fortessa (BD Bioscience).

#### NK cell lysosomal acidification.

PBMCs were incubated for 30–45 minutes at 37°C, with LysoTracker (Invitrogen) and LysoSensor (Invitrogen) probes to assess lysosomal number and pH, respectively. PBMCs stained with LysoTracker and LysoSensor were analyzed on LSR Fortessa (BD Bioscience).

#### NK cell cytokine production and degranulation.

PBMCs were stimulated with or without cytokines (IL-2 and IL-12, 50 ng/mL and 20 ng/mL respectively) for 6 hours at 37°C. BD GolgiPlug, BD GolgiStop, and CD107a-PE were added 4 hours before readout. After incubation, cells were stained with Live/Dead-APC-H7 and cell surface antibodies: CD3-BUV737 (clone UCHT1; BD Biosciences; catalog no 612750), CD4-PB (clone RPA-T4; BD Biosciences; catalog no 558116), CD8-BV605, (clone SK1; BD Biosciences; catalog no 564116), CD19-FITC (clone REA675; Miltenyi no 130-113-645), and CD56-BUV395 (clone NCAM16.2; BD Biosciences; catalog no 563554). After permeabilization with BD Cytofix/Cytoperm kit, cells were stained with IFN-γ–AF700 (clone B27; BD Biosciences; catalog no 557995) and TNF-α–APC (clone Mab11; BD Biosciences; catalog no 551384) before analysis on LSR Fortessa (BD Biosciences).

### HCQ and Urolithin A

NK cells or PBMCs from HC and patients with SLE were incubated overnight at 37°C, in presence of HCQ sulfate (5 mg) or Urolithin A (5 mg), purchased from Sigma. PBMCs were treated with 100nM of HCQ sulfate or 1 μM of Urolithin A whereas isolated NK cells were stimulated with 1 μM of HCQ sulfate or 10 μM of Urolithin A.

### NK cell isolation

NK cells were enriched from SLE and healthy PBMCs using the human NK isolation kit (Miltenyi) with negative labeling. The labeled PBMCs were processed through the AutoMACS ProSeparator (Miltenyi Biotec).

### NK cell cytosol/mitochondria fragmentation

NK cells were isolated from SLE and HC PBMCs. Cytosolic, mitochondrial, and whole-cell extract fractions were separated using the cytosol/mitochondria fragmentation kit (Abcam) according to the manufacturer’s instructions. Briefly, isolated NK cells were washed in PBS and pelleted by centrifugation at 600*g* for 5 minutes. The cell pellet was resuspended in the cytosol extraction buffer and incubated on ice for 10 minutes, followed by homogenization with a syringe. The homogenate was centrifuged at 1,000*g* for 10 minutes, and the supernatant, containing the cytosolic fraction, was collected. This supernatant was further centrifuged at 10,000*g* for 30 minutes. Both supernatant and cell pellet resulting from the last centrifugation were saved as cytosolic fraction and mitochondria, respectively. The mitochondria were resuspended in the mitochondrial extraction buffer and saved as the mitochondrial fraction.

The whole cell extract was obtained by centrifugating NK cells at the initial step, without fractionation. DNA was extracted from all the obtained fractions (cytosolic, mitochondrial, whole cell) using the QIAamp DNA Mini Kit (Qiagen) according to the manufacturer’s instructions.

### Quantification of mtDNA content

The mtDNA content in different NK subcellular fractions was quantified using qPCR targeting NADH deshydrogenase 2 (*ND2*) and *D-Loop* (displacement loop). Amplifications were performed using PowerTrack SYBR Green and TaqMan Universal PCR Master Mixes (Life Technologies Europe BV). Data were acquired with the QuantStudio 6 Flex Real-Time PCR System (ThermoFisher Scientific). Quantification of the mtDNA content in the cytosol of healthy and SLE NK cells was assessed following the basic protocol 2 as previously described (18). Results were normalized to Alu repeat sequence. Primers for qPCR were ordered from Microsynth and are detailed in Supplemental Methods.

### Mitophagy genes expression

RNA was extracted from NK cells of SLE and HC using the RNeasy Plus Mini Kit (Qiagen) according to the manufacturer’s instructions. Complementary DNA was synthesized, and qPCR was performed with SuperScript III Platinum SYBR Green One-Step qRT-PCR Kit (Life Technologies). The expression of mitophagy-related genes including light chain 3B (*LC3B*), Lysosome-associated membrane protein 2 (*LAMP2*), PTEN Induced Kinase 1 (*PINK1*), Parkinson disease 2 (*PARK2*), class III phosphatidylinositol 3-kinase (PIKC3C), GABA Type A Receptor Associated Protein Like 1 (*GABARAPL1*), Unc-51-like kinase 1 (*ULK1*), a nd Beclin 1 (*BECN1*) genes was assessed using the QuantStudio 6 Flex Real-Time PCR System (ThermoFisher Scientific). Results were normalized to the expression β-actin. Primers were ordered from Microsynth (Supplemental Methods).

### TEM

TEM was performed on NK cells isolated from the peripheral blood of SLE (*n* = 5) patients and HC (*n* = 5). Detailed procedures are provided in the online supplemental materials.

### Proteomics analyses

Liquid chromatography–tandem mass spectrometry (LC-MS/MS) analyses were performed on NK cells isolated from the peripheral blood of patients with SLE (*n* = 4) and HC (*n* = 4). Detailed procedures are provided in the online supplemental materials. All raw MS data, along with processed raw output tables, are publicly available via the ProteomeXchange data repository (www.proteomexchange.org) with the accession code PXD059825.

### Statistics

Statistical analyses were performed using GraphPad Prism (version 10). Details of the statistical tests and sample sizes for each experiment are provided in the corresponding figure legends.

For comparisons between 2 groups with nonnormal distributions and paired data sets the Mann-Whitney *U* test was used. For comparisons between 2 groups with nonnormal distributions and not paired data sets, the Wilcoxon signed-rank test was performed. For comparisons between 2 groups with normal distributions, paired or unpaired, 2-tailed Student’s *t* tests were used as appropriate. Normality was assessed via the Shapiro-Wilk test. For multiple-group comparisons with nonnormal distributions, the Kruskal-Wallis test was applied, with *P* values adjusted using Dunn’s method. For multiple-group comparisons with normal distributions, 1-way ANOVA was used, with *P* values adjusted using the Šídák’s method. For multiple-group comparisons with normal distributions and not paired data sets, mixed-effects analysis was used, with *P* values adjusted using the Turkey’s method. Two-way ANOVA was used for comparisons involving 2 independent variables, with *P* values adjusted using Šídák’s or Tukey’s methods as appropriate. *P* < 0.05 was considered statistically significant. Data are shown as mean ± SEM.

### Study approval

Written Informed consent was obtained from all individuals, and the study was approved by the IRB of the Cantonal Ethics Committee of Vaud (CER-VD), Lausanne, Switzerland (SwissEthics 2017-01434), in compliance with the Declaration of Helsinki.

### Data availability

The raw data supporting the conclusions of this article will be made available by the authors, without undue reservation. Supporting data values associated with the figures, including all individual data points and values underlying reported means, are provided in the Supporting Data Values file.

## Author contributions

NF and DC contributed the following: study design and data analysis. NF, MH, and ER contributed the following: conducting experiments, data acquisition and analysis. NF, CR, and DC contributed the following: recruitment of patients with SLE and HC. NF and DC contributed the following: writing and editing of manuscript. MH, GT, ER, AAJ, and CR contributed the following: critical reading of the manuscript. All authors contributed to the article and approved the submitted version.

## Funding support

This work is the result of NIH funding, in whole or in part, and is subject to the NIH Public Access Policy. Through acceptance of this federal funding, the NIH has been given a right to make the work publicly available in PubMed Central.

- Swiss National Science Foundation Ambizione PZ00P3_173950 (DC).
- Novartis Foundation 19A022 (DC).
- Swiss National Science Foundation 310030_200796 (AAJ).
- NIH (PHS R01AI148161 to GT.)

## Supplementary Material

Supplemental data

Supporting data values

## Figures and Tables

**Table 1:**
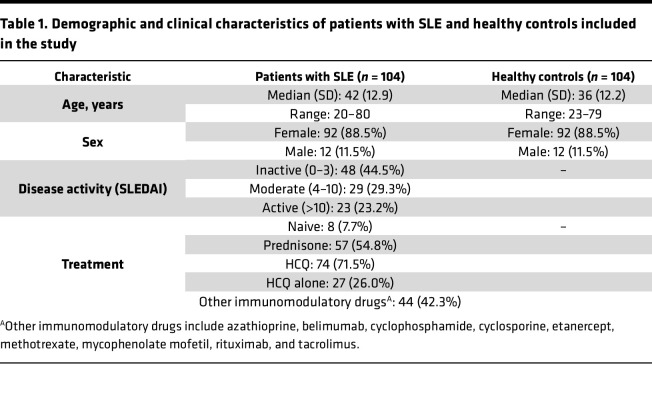
Demographic and clinical characteristics of patients with SLE and healthy controls included in the study

## Version 1 02/02/2026

In-Press Preview

## Version 2 03/09/2026

Electronic publication

## Version 3 04/21/2026

Corrected a technical issue with link to supplemental material
