# Supplementary material for: Mitochondrial dysfunction drives natural killer cell dysfunction in systemic lupus erythematosus
Source: JCI Insight. 2026 Jan 29;11(5):e195170. doi: 10.1172/jci.insight.195170 (PMC13041687; doi:10.1172/jci.insight.195170)
Supplement: Supplementary file 1 [file jciinsight-11-195170-s310.pdf]

SUPPLEMENTAL MATERIAL

Mitochondrial dysfunction drives natural killer cell dysfunction in systemic lupus erythematosus

Natalia W. Fluder[1], Morgane Humbel[2], Emeline Recazens[3], Alexis A. Jourdain[3], Camillo Ribi[1], George C. Tsokos[2], Denis Comte[4]

Affiliations:

[1] Department of Medicine, Division of Immunology and Allergy, Lausanne University Hospital, University of Lausanne, 1011 Lausanne, Switzerland.

[2] Department of Medicine, Beth Israel Deaconess Medical Center, Harvard Medical School, Boston, USA.

[3] Department of Immunobiology, University of Lausanne, 1066 Epalinges, Switzerland.

[4] Department of Medicine, Division of Internal Medicine, Lausanne University Hospital, University of Lausanne, 1011 Lausanne, Switzerland.

SUPPLEMENTAL METHODS

Transmission electron microscopy (TEM) Cells were centrifuged at approximately 700 × g (corresponding to ~2500 rpm in a standard benchtop centrifuge), the supernatant was removed and the cells were then fixed in 2.5% glutaraldehyde solution (EMS, Hatfield, PA) and in osmium tetroxide 1% (EMS) with 1.5% of potassium ferrocyanide (Sigma, St. Louis, MO) in phosphate buffer (PB 0.1 M [pH 7.4]) for 1h at room temperature. The samples were then centrifuged at approximately 2800 × g (corresponding to ~5000 rpm), the supernatant was removed and replaced with distilled water twice. Then the cells were centrifuged again at approximately 2800 × g (corresponding to ~5000 rpm) and the supernatant removed. After that, the cells were dehydrated in acetone solution (Sigma, St Louis, MO, USA) at graded concentrations (30%-15 min; 70% - 15 min; 100% - 4x15). This was followed by infiltration in Epon resin (EMS, Hatfield, PA, US) at graded concentrations (Epon 33% in acetone-2h; Epon 66% in acetone-4h; Epon 100%-2x8h) and finally polymerized for 48h at 60°C in an oven. Ultrathin sections of 50 nm were cut using a Leica UC7 (Leica Mikrosysteme GmbH, Vienna, Austria), picked up on a copper slot grid 2x1mm (EMS, Hatfield, PA, US) coated with a polystyrene film (Sigma, St Louis, MO, USA). Sections were post-stained with uranyl acetate (Sigma, St Louis, MO, USA) 4% in H2O for 10 min, rinsed several times with H2O followed by Reynolds lead citrate in H2O (Sigma, St Louis, MO, USA) for 10 min and rinsed several times with H2O. Micrographs were taken with a transmission electron microscope FEI CM100 (FEI, Eindhoven, The Netherlands) at an acceleration voltage of 80kV with a TVIPS TemCamF416 digital camera (TVIPS GmbH, Gauting, Germany).

Note: Relative centrifugal force (× g) values were estimated from the originally reported rpm, as the exact centrifuge model and rotor radius were not available

Proteomic analyses Sample preparation and protein digestion: Washed cell pellets (3-5 10^5 cells) were lysed in 25 µl miST lysis buffer (1% Sodium deoxycholate, 100mM Tris pH 8.6, 10 mM DTT) and heated for 10min at 75°C. Proteins were digested following a modified version of the iST method (Kulak, et al., 2014) (named miST method). Based on tryptophane fluorescence quantification (Wisniewski, et al., 2015), approximately 30 ug of proteins at 1µg/µl were transferred to new tubes, diluted 1:1 (v:v) with water containing 4mM $MgCl_2$ and benzonase (Merck #70746, 100x dil of stock = 250 Units/µl), and incubated for 15 minutes at room temperature to digest

nucleic acids. Reduced disulfides were alkylated by adding ¼ vol. of 160 mM chloroacetamide (32 mM final) and incubating for 45min at room temperature in the dark. Samples were adjusted to 3 mM EDTA and digested with 0.5 µg Trypsin/LysC mix (Promega #V5073) for 1h at 37°C, followed by a second 1h digestion with an additional 0.5µg of proteases. To remove sodium deoxycholate, two sample volumes of isopropanol containing 1% TFA were added to the digests, and the samples were desalted on a strong cation exchange (SCX) plate (Oasis MCX; Waters Corp., Milford, MA) by centrifugation. After washing with isopropanol 1%TFA, peptides were eluted in 200µl of 80% MeCN, 19% water, 1% (v/v) ammonia, and dried by centrifugal evaporation.

Liquid Chromatography-Mass Spectrometry analyses LC-MS/MS analyses were carried out on a TIMS-TOF Pro (Bruker, Bremen, Germany) mass spectrometer interfaced through a nanospray ion source ("captive spray") to an Ultimate 3000 RSLCnano HPLC system (Dionex). Peptides were separated on a reversed-phase custom packed 45 cm C18 column (75 µm ID, 100Å, Reprosil Pur 1.9 um particles, Dr. Maisch, Germany) at a flow rate of 250 nl/min with a 2-27% acetonitrile gradient in 93 min followed by a ramp to 45% in 15 min and to 90% in 5 min (total method time: 140 min, all solvents contained 0.1% formic acid). Approximately 0.25 µg of digest were injected.

The data-independent acquisition (DIA) used mostly the instrument parameters reported previously (Meier, et al. 2020). Per cycle, the mass range 400-1200 m/z was covered by a total of 32 windows, each 25 Th wide and a 1/k0 range of 0.3. Collision energy was ramped linearly based uniquely on the 1/k0 values from 20 (at 1/k0=0.6) to 59 eV (at 1/k0=1.6). Two windows were acquired per TIMS scan (100ms) so that the total cycle time was 1.7 s.

Data processing Raw Bruker MS DIA data were processed directly with Spectronaut 16.1 (Biognosys, Schlieren, Switzerland) with the Pulsar engine using the "deep" setting and searching the human SWISSPROT database (www.uniprot.org) of January 7th, 2022 (20'375 sequences). For identification, peptides of 7-52 AA length were considered, cleaved with trypsin/P specificity and a maximum of 2 missed cleavages. Carbamidomethylation of cysteine (fixed), methionine oxidation and N-terminal protein acetylation (variable) were the modifications applied. FDR's for peptide and protein group identifications were all at 1%. Ion mobility for peptides was predicted using a

deep neural network and used in scoring. The library created contained overall 91’819 precursors (71’900 peptides).

Peptide-centric analysis of DIA data was done with Spectronaut 16.1 using the library generated by Pulsar from DIA data. Single hits proteins (defined as matched by one stripped sequence only) were kept in the Spectronaut analysis. Peptide quantitation was based on XIC area, for which a minimum of 1 and a maximum of 3 (the 3 best) precursors were considered for each peptide, from which the median value was selected. Quantities for protein groups were derived from inter-run peptide ratios based on MaxLFQ algorithm (Cox et al 2014). Global normalization of runs/samples was done based on the median of peptides. Overall, 6’360 protein groups were identified at 1% FDR.

Data analysis All subsequent analyses were done with the Perseus software package (version 1.6.15.0) (Tyanova et al 2016). Quantities were log2-transformed and contaminants removed. After assignment to conditions, only proteins quantified in at least 2 samples of one condition were kept (6’256 protein groups). After missing values imputation (based on normal distribution using Perseus default parameters), t-tests were carried out among all conditions, with permutation-based FDR correction for multiple testing (Q-value threshold <0.05). Imputed values were later removed. The difference of means obtained from the tests were used for 1D enrichment analysis on associated GO/KEGG annotations as described (Cox and Mann, 2012). The enrichment analysis was also FDR-filtered (Benjamini-Hochberg, Q-val<0.02).

Supplemental Table 1: Characteristics of SLE patients included in the study.

FIGURE	SAMPLE	SLEDAI	TREATMENT	YOB	SEX
Fig 1. A, B, C, D	SLE 001	4	HCQ, Beli	1970	F
	SLE 002	3	AZA	1963	F
	SLE 003	14	NA	1954	F
	SLE 004	6	PDN5, HCQ, Beli, RTX, CIA, MTX, IVIg	1993	F
	SLE 005	2	HCQ	1965	F
	SLE 006	8	PDN2.5, HCQ	1965	F
	SLE 007	2	AZA, CYC, RTX, IVIg	1969	F
	SLE 008	25	PDN17.5, HCQ, Beli, MMF	1982	F
	SLE 009	5	No treatment	2000	M
	SLE 010	12	HCQ	1996	M
	SLE 011	18	PDN8, HCQ, MMF, Beli	1992	F
	SLE 012	4	PDN5, HCQ, Beli	1983	F
	SLE 013	10	HCQ	1944	F
	SLE 014	11	NSAIDS	1972	F
	SLE 015	7	HCQ	1991	F
	SLE 016	0	HCQ	1970	F
	SLE 017	2	PDN5	1948	F
	SLE 018	24	PDN50, NSAIDS	2000	F
Fig 1. F	SLE 019	4	PDN2.5, HCQ	1970	F
	SLE 020	0	PDN2.5, HCQ	1980	F
	SLE 021	6	PDN30, HCQ	1996	F
	SLE 022	8	HCQ	1980	F
	SLE 023	22	HCQ	1973	F
Fig 1. G	SLE 024	37	PDN, HCQ	1986	F
	SLE 025	2	HCQ	1980	F
	SLE 026	0	PDN, HCQ, MMF	1994	F
	SLE 027	3	Beli	1973	F
	SLE 028	3	HCQ	1984	M
	SLE 029	13	PDN, HCQ, MMF	1996	F
	SLE 030	2	HCQ	1965	F
	SLE 031	4	PDN, HCQ	1956	F
	SLE 032	18	PDN, HCQ, MMF	1972	F
	SLE 033	2	HCQ	1991	F
	SLE 034	2	HCQ	1982	F
	SLE 035	0	HCQ	1985	F
	SLE 036	8	HCQ	1980	F
	SLE 037	8	PDN, HCQ	1972	F
	SLE 038	23	No treatment	1990	F
	SLE 039	24	PDN	2000	F

Fig 2. A, B, C	SLE 040	2	No treatment	1991	F
	SLE 041	0	No treatment	1972	F
	SLE 042	4	HCQ	1979	F
	SLE 043	11	PDN7.5, HCQ	1990	F
Fig 2. D, E, Fig 3. A	SLE 044	0	HCQ	1973	F
	SLE 045	4	PDN10, HCQ	1975	M
	SLE 046	8	PDN15, HCQ	1980	F
	SLE 047	0	PDN5, HCQ	1981	F
	SLE 048	0	HCQ	1991	F
	SLE 049	6	PDN5, HCQ, Beli	1997	F
	SLE 050	20	No treatment	1980	M
	SLE 051	10	PDN7.5, HCQ, MTX	1976	F
	SLE 052	NA	NA	NA	NA
	SLE 053	4	PDN5, HCQ, MMF	1985	M
	SLE 054	0	HCQ, MTX	1995	F
	SLE 055	26	PDN40, HCQ, CYC	2000	F
	SLE 056	3	Beli	1973	F
	SLE 057	2	PDN5, HCQ, Beli	1988	F
	SLE 058	26	PDN40, HCQ, CYC	2000	F
	SLE 059	0	PDN7.5, HCQ, MTX, Beli	1996	F
	SLE 060	7	PDN15, HCQ, AZA, Beli	1976	F
	SLE 061	1	HCQ	1970	F
	SLE 062	20	No treatment	1980	M
	SLE 063	NA	NA	NA	NA
	SLE 064	7	Colchicine	1975	M
	SLE 065	2	PDN5, HCQ, Beli	1997	F
	SLE 066	6	PDN5, HCQ, Beli	1997	F
	SLE 067	30	Etanercept	1985	M
	SLE 068	6	PDN5, HCQ, Beli	1993	F
	SLE 069	6	PDN7.5, CYC	1972	F
	SLE 070	2	No treatment	1957	F
	SLE 071	0	HCQ	1973	F
	SLE 072	0	HCQ	1975	F
	SLE 073	37	PDN40, HCQ	1986	F
	SLE 074	5	PDN5, HCQ	1982	F
	SLE 075	0	PDN5, HCQ, MMF	1978	F
	SLE 076	12	PDN12.5, MMF	1992	F
	SLE 077	23	PDN7.5, HCQ, AZA	1972	F
Fig 3. C, D	SLE 078	6	PDN5, HCQ, Beli	1983	F
	SLE 079	0	HCQ, Beli	1970	F
	SLE 080	0	PDN5, HCQ	1935	F

	SLE 081	0	PDN5, HCQ	1973	M
	SLE 082	2	Beli	1963	F
	SLE 083	0	PDN5, MMF	1973	F
	SLE 084	4	PDN5, HCQ, Beli, MMF	1979	F
	SLE 085	2	HCQ, Beli	1965	F
	SLE 086	6	PDN5, HCQ, BEN	1983	F
	SLE 087	2	RTX, HCQ	1981	F
	SLE 088	0	HCQ	1976	F
	SLE 089	0	PDN5, HCQ, Beli	1968	F
	SLE 090	3	No treatment	1962	F
	SLE 091	2	PDN5, MTX, Beli	1980	F
	SLE 092	12	PDN50, HCQ	1984	F
Fig 3. E, Fig 6.	SLE 093	2	PDN5, HCQ	1972	F
	SLE 094	0	HCQ	1962	F
	SLE 095	2	PDN4, HCQ	1982	F
	SLE 096	6	PDN5, HCQ, Beli	1983	F
	SLE 097	4	PDN10, HCQ	1988	F
	SLE 098	8	PDN20, HCQ, Beli	1988	F
	SLE 099	0	HCQ, Beli	1965	F
	SLE 100	0	HCQ	1990	F
	SLE 101	0	No treatment	1993	F
	SLE 102	8	PDN5, HCQ, Beli	1972	F
	SLE 103	4	PDN7.5, HCQ, Beli	1963	F
	SLE 104	6	PDN5, HCQ, MTX, Beli	1979	F
	SLE 105	0	HCQ	1990	F
	SLE 106	0	HCQ	1976	F
	SLE 107	2	PDN2.5, HCQ	1962	F
	SLE 108	NA	PDN7, HCQ, Beli	1982	F
Fig 4. A, B, C, D, E, F, Fig 5. A, B, C, D, E	SLE 109	10	PDN5, HCQ, MTX, Beli	1979	F
	SLE 110	17	No treatment	1999	F
	SLE 111	5	PDN10, HCQ	1980	M
	SLE 112	3	PDN5, MTX	1980	F
	SLE 113	0	HCQ, CIA	1982	F
	SLE 114	0	PDN5, HCQ, MTX	1991	F
	SLE 115	0	PDN5, HCQ, MMF	1985	M
	SLE 116	0	HCQ, Beli	1970	F
	SLE 117	4	PDN5, HCQ, Beli	1940	M
	SLE 118	0	HCQ	1973	F
	SLE 119	2	HCQ	1981	F
	SLE 120	0	Beli	1973	F
	SLE 121	NA	NA	NA	NA

	SLE 122	0	No treatment	1979	F
	SLE 123	2	NA	1984	M
	SLE 124	0	HCQ, Beli, MTX	1978	F
	SLE 125	0	HCQ	1955	F

Healthy controls were matched to patients for sex and ethnicity and were age-matched within ±10 years. Abbreviation: PDN, prednisone; HCQ, hydroxychloroquine; Beli, belimumab, AZA, azathioprine, MMF, mycophenolate mofetil; MTX, methotrexate; CYC, cyclophosphamide; RTX: rituximab; CIA, cyclosporin A; IVIg, intravenous immunoglobulin; NSAIDS, nonsteroidal anti-inflammatory drugs; NA, indicates data not available from retrospective clinical records.

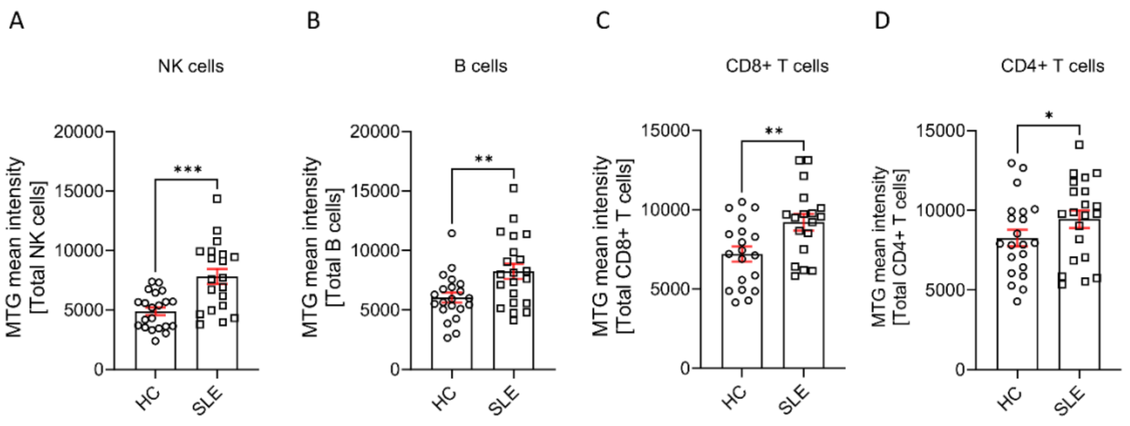

<u>Supplemental figure 1: Comparison of mitochondrial mass among PBMC populations from SLE patients and healthy controls.</u>

Mitochondrial mass in NK cells (A), B cells (B), CD8+ T cells (C), CD4+T cells (D) from HC (n=19) and SLE patients (n=19), assessed by flow cytometry using MitoTracker Green (MTG) after overnight resting. Each symbol represents one individual. *p<0.05, by Wilcoxon test.

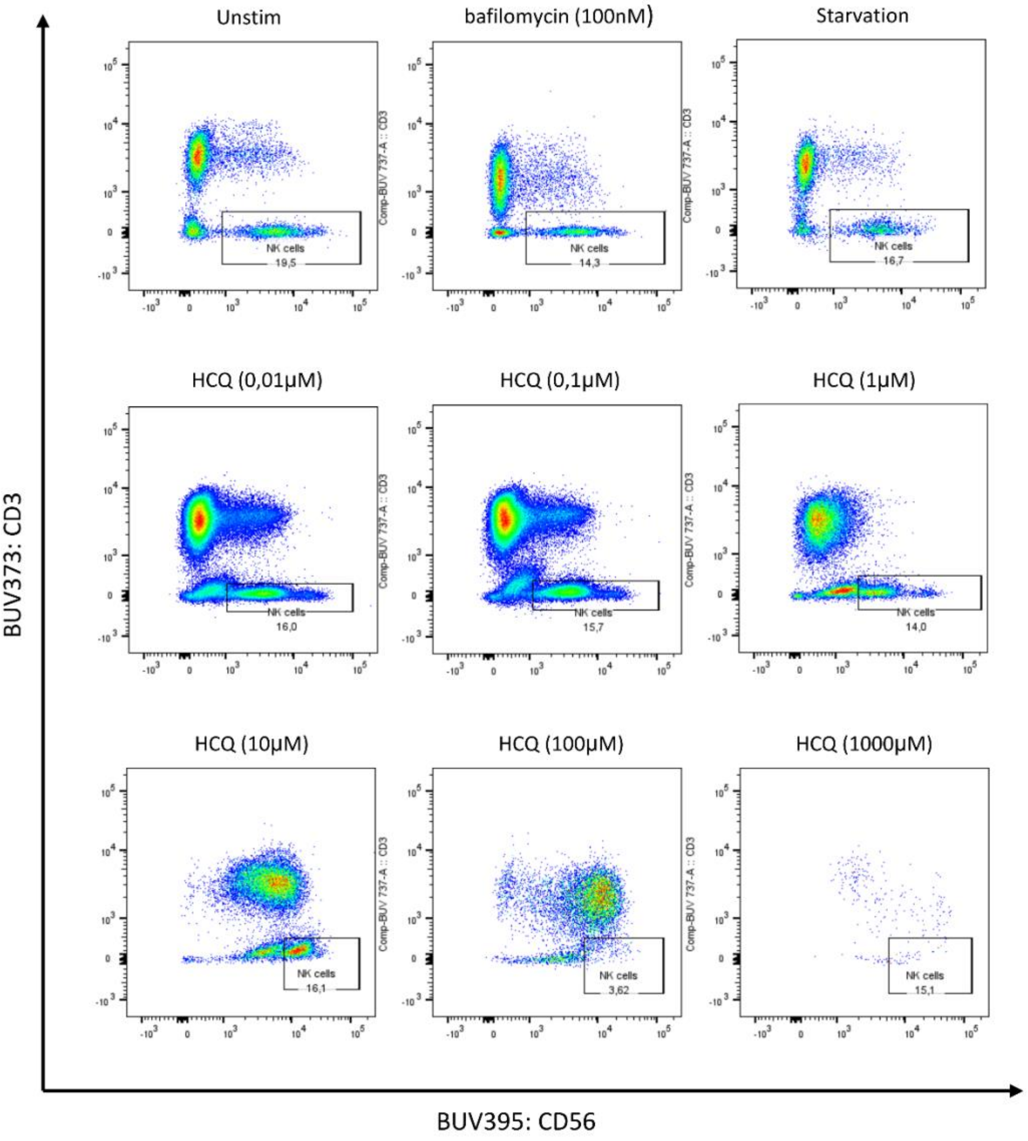

Supplemental figure 2: Impact of hydroxychloroquine concentration on NK cells phenotype alteration in healthy controls.

Representative flow cytometry dot plots showing gating strategy for NK cells ($CD3^{-}CD56^{+}$) during assay optimization. Lysosomal number was assessed by flow cytometry based on LysoTracker mean fluorescence intensity (MFI). Peripheral blood mononuclear cells (PBMCs) from healthy controls were stimulated overnight in the presence or absence of bafilomycin A1 (100 nM), increasing concentrations of hydroxychloroquine (HCQ; 0.01, 0.1, 1, 10, 100, and 1000 µM), or under starvation conditions.

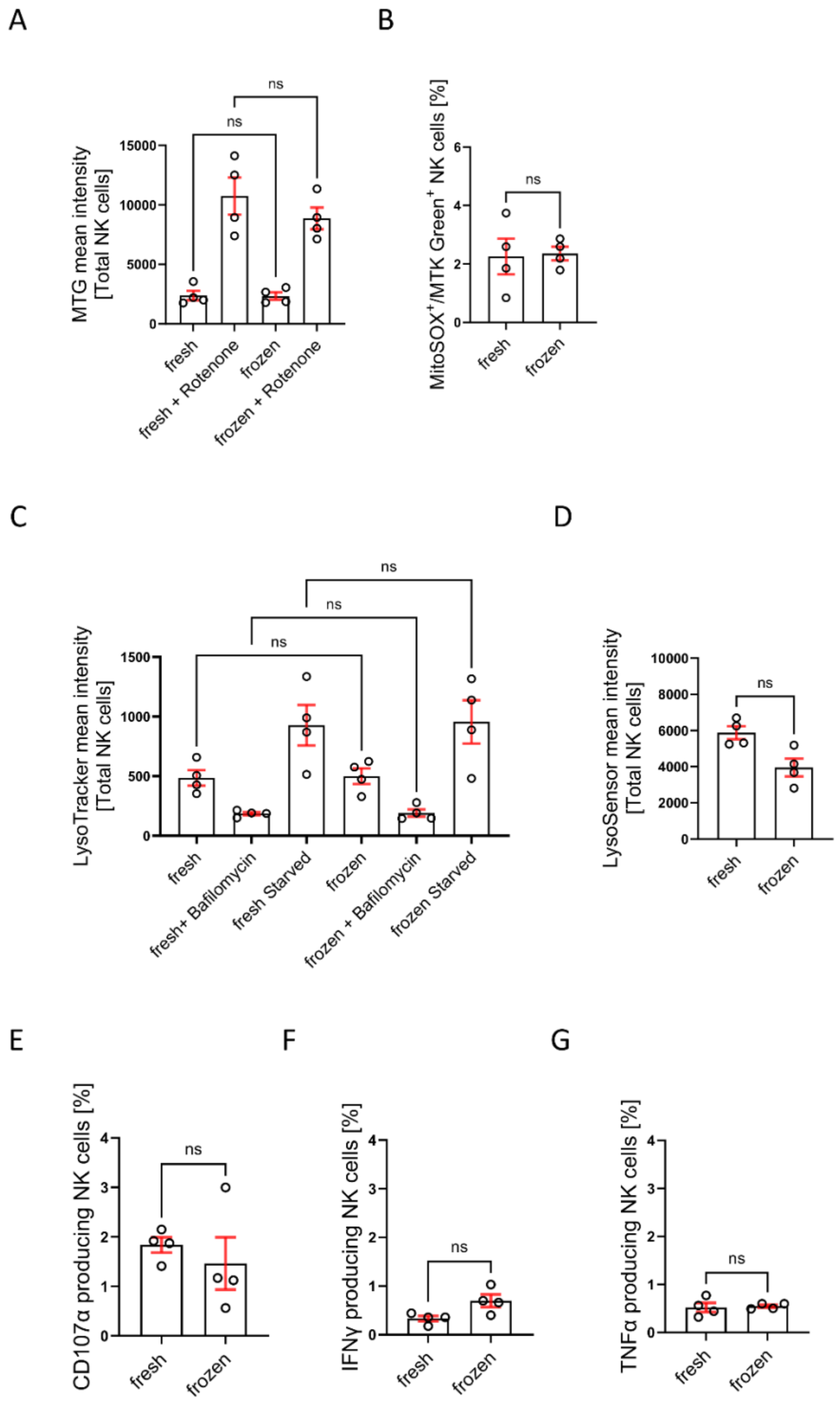

<u>Supplemental figure 3: Comparable NK cells function and phenotype in freshly isolated versus frozen PBMCs.</u>

Freshly isolated or thawed peripheral blood mononuclear cells (PBMCs) from healthy controls (HC) were rested overnight prior to analysis.

(A) Mitochondrial mass in NK cells assessed by flow cytometry using MitoTracker Green mean fluorescence intensity (MFI). *p<0.05 by Wilcoxon test.

(B) Mitochondrial superoxide levels assessed by flow cytometry based on the frequency of MitoSOX$^+$/MitoTracker Green$^+$ NK cells. Rotenone was used as a positive control. *p<0.05 by paired t test.

(C, D) Lysosomal number (C) and lysosomal pH (D) in NK cells assessed by flow cytometry using LysoTracker and LysoSensor MFI, respectively. Bafilomycin A1 and starvation were used as negative and positive controls, respectively. *p<0.05 by Wilcoxon test.

(E–G) NK cell function assessed after overnight resting. Degranulation (E) and cytokine production (F, G) were measured by flow cytometry based on the frequency CD107α+ (E), IFNγ+ (F), and TNFα+ (G) cells. *p<0.05, **p<0.01, ***p<0.001, by Kruskal-Wallis test

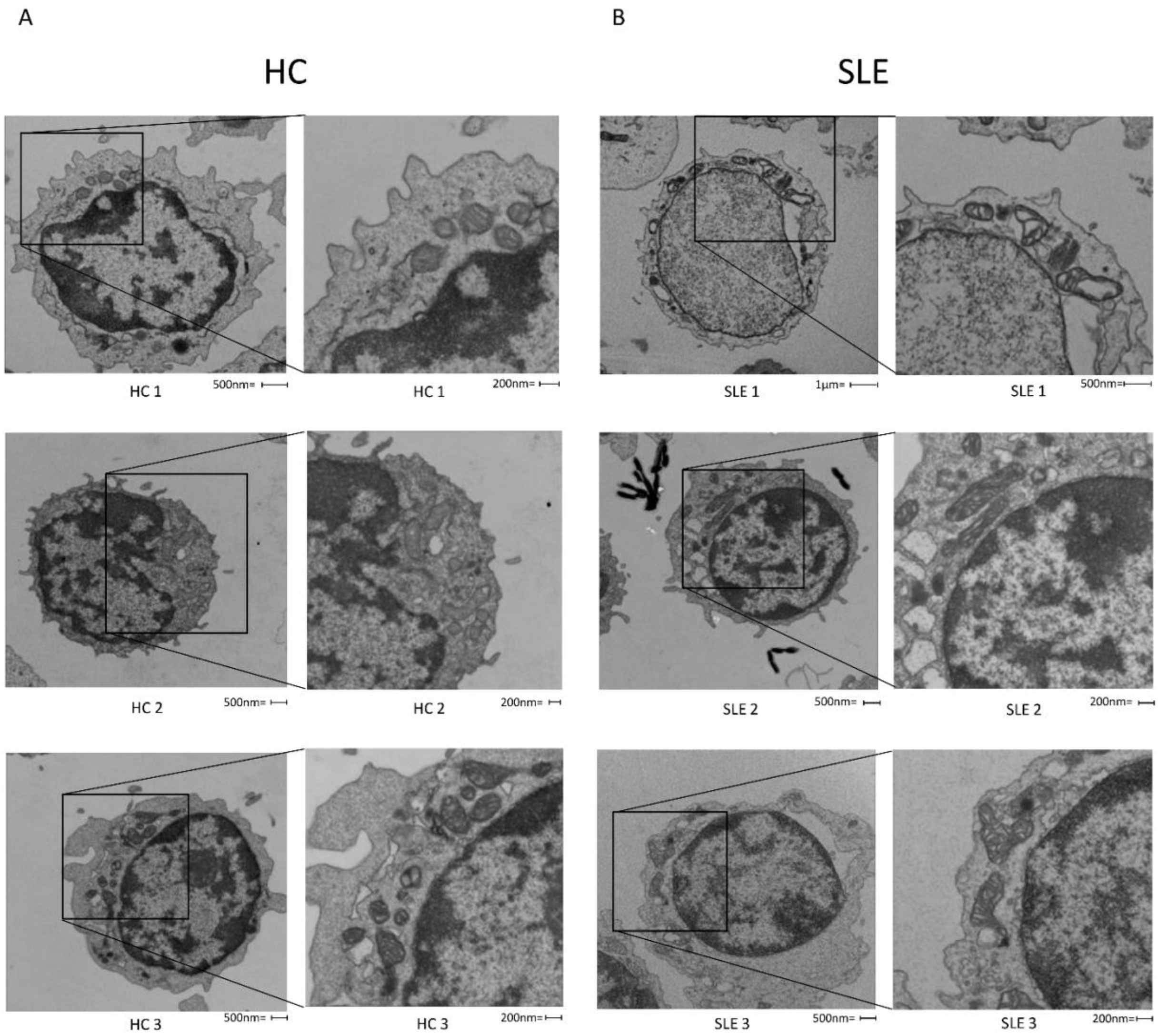

<u>Supplemental figure 4: TEM comparison of mitochondrial ultrastructure in NK cells from healthy controls and SLE patients.</u>

(A, B) Transmission electron microscopy (TEM) images of NK cells isolated from healthy controls (HC) (A) and SLE patients (B). Representative images illustrate mitochondrial ultrastructure within NK cells from each group.

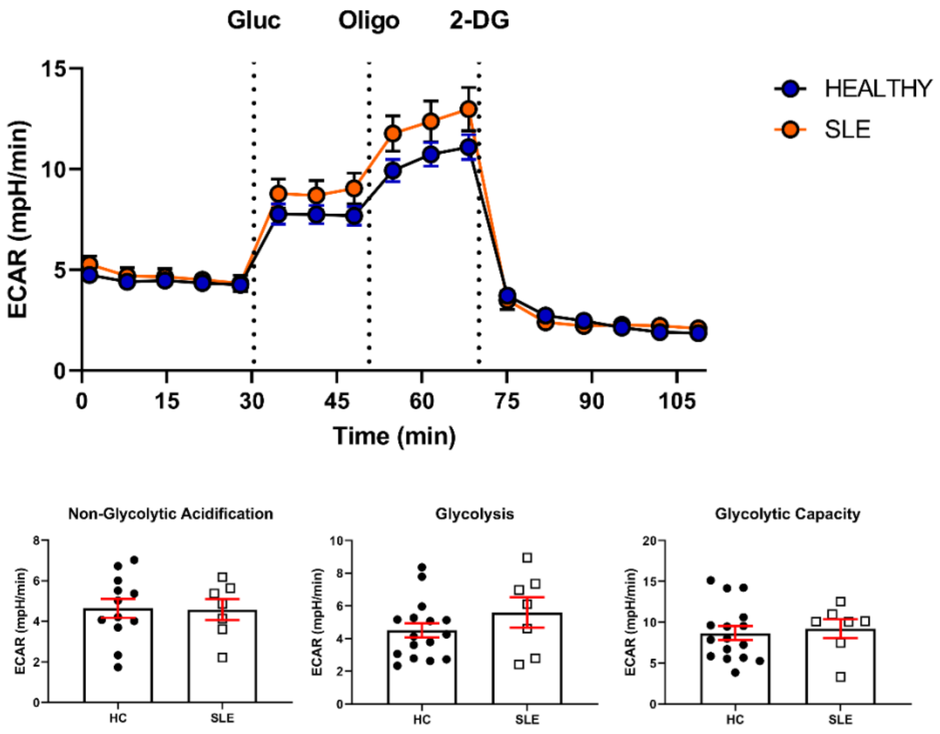

Supplemental figure 5: Exploratory Seahorse analysis of glycolytic flux (ECAR) in primary NK cells from healthy controls and SLE patients.

Extracellular acidification rate (ECAR) was measured over time in primary NK cells from healthy controls (HC, n=13) and SLE patients (n=7). Dotted lines indicate sequential injections of glucose (Glc), oligomycin (Oligo), and 2-deoxy-D-glucose (2-DG). Baseline ECAR reflects non-glycolytic acidification, whereas glucose and oligomycin injections allow assessment of glycolysis and glycolytic capacity, respectively. $*p<0.05$, $**p<0.01$, $***p<0.001$ by Welch’s test.
